# Coronary Sinus to Left Atrial Communication

**DOI:** 10.1155/2009/790715

**Published:** 2009-08-26

**Authors:** Vandhana Scheller, Wojciech Mazur, James Kong, Eugene S. Chung

**Affiliations:** Ohio Heart and Vascular Center, The Christ Hospital, Cincinnati, OH 45242, USA

## Abstract

Congenital coronary sinus anomalies are rare in clinical practice, partly due to the lack of symptoms. We present a case of coronary sinus anomaly causing a right-to-left intracardiac shunt in a 46 years/old African American female with a past medical history of obstructive sleep apnea, diabetes mellitus, hypertension, coronary artery disease, and ischemic cardiomyopathy who presented with hypoxia. In the months prior to her presentation, she had suffered an inferior myocardial infarction with right ventricular involvement, as well as resulting severe tricuspid regurgitation. In conclusion, further investigations revealed a communication between the coronary sinus (CS) and left atrium (LA).

## 1. Case Description

**Figure 1:**
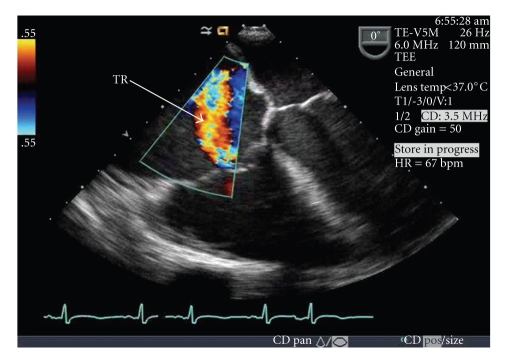
TEE showing severe tricuspid regurgitation.

 A 46 years/old African American female was admitted to an outside hospital where she initially presented with shortness of breath and hypoxia. She had a history of right coronary artery stenting several years ago surrounding an inferior myocardial infarction possibly with right ventricular involvement and relatively preserved left ventricular (LV) ejection fraction. Her follow-up and compliance had been suboptimal in the interim period. Initially, she had a peripheral oxygen saturation of 77% with a pAO_2_ of 47 mmHg. She showed no evidence of an acute coronary syndrome, and a head CT performed for history of falls demonstrated no significant abnormalities. A shunt study in the form of ventilation perfusion scan revealed early uptake of radioisotope in the brain, suggestive of a right-to-left shunting. Subsequently, a transesophageal echocardiogram revealed a severe right-to-left shunt with immediate appearance of bubbles in the left side although no apparent atrial or ventricular septal defect was seen. LV ejection fraction was moderately depressed, and the right ventricle and atrium were noted to be dilated. Severe tricuspid regurgitation was described. (Figure 1) She was then transferred to The Christ Hospital, Cincinnati, Ohio, for further evaluation in the cardiac catheterization laboratory for a presumed occult atrial septal defect and possible percutaneous closure. 

Upon admission to our institution vital signs were stable, and physical exam was pertinent for elevated jugular venous pressure and severe lower extremity pitting edema. She was 5′2″ and 285 lbs on admission. Heart sounds were normal with only a soft holosystolic murmur. Electrocardiogram showed sinus rhythm with inferior Q waves and no acute changes. Evaluation in the cardiac catheterization laboratory revealed the following hemodynamic profile: right atrium (RA) 25 mmHg, right ventricle (RV) 45/25 mmHg, pulmonary artery (PA) 45/20 mmHg, pulmonary capillary wedge pressure (PCWP) 17 mmHg. There was no oxygen saturation step-up between RA to RV or the PA. Intracardiac echocardiography demonstrated an intact interatrial septum and numerous attempts to find a defect using catheters were unsuccessful. Coronary angiography showed patent right coronary artery (RCA) stent and minimal disease in the left coronary system. 

**Figure 2:**
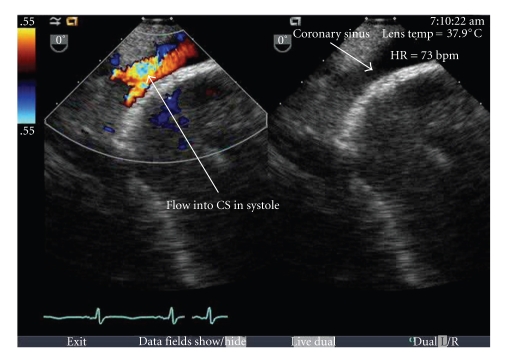
TEE showing doppler signal away from LA. The arrows indicate a dilated coronary sinus.

**Figure 3:**
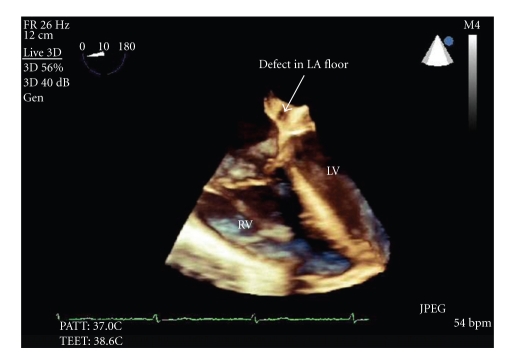
TEE. The left arrow indicates the interatrial septum, and the right arrow indicates the opening at the inferoposterior corner of the LA floor.

**Figure 4:**
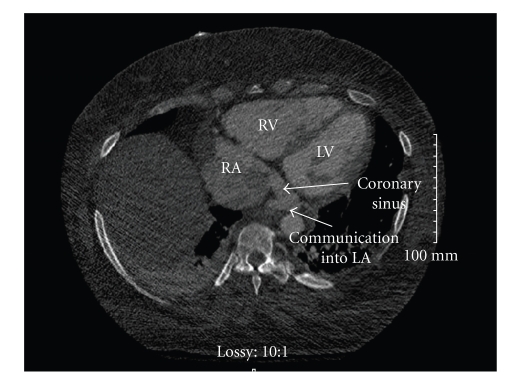
Cardiac CTA. The arrows indicate the coronary sinus with a communicating vessel to the LA.

 Her oxygen requirements were only 2-3 L/min by nasal cannula at baseline, to maintain oxygen saturation of 93%. To relieve discomfort due to her severe volume overload, and as the target volume was deemed to be in excess of 20 lbs, she was started on ultrafiltration using the Aquadex system (CHF Solutions, Inc.), with eventual removal of approximately 25 lbs of weight. With this degree of volume removal, it was noted that the patient's oxygen requirement appeared to increase to 8 L/minute oxygen via oximizer. A repeat transesophageal echocardiogram (TEE) was performed confirming the findings of the outlying hospital. Furthermore, on this study, a “ridge” at the inferior aspect of the interatrial septum was noted associated with a color Doppler signal away from the RA. (Figure 2) A 3-dimensional assessment of the LA suggested a small opening at the inferoposterior corner. (Figure 3) A cardiac CT angiogram demonstrated moderate cardiomegaly with a small pericardial effusion; however, a severely dilated coronary sinus was detected, possibly with an “unroofed” appearance, with apparent communication into the LA. (Figure 4).

**Figure 5:**
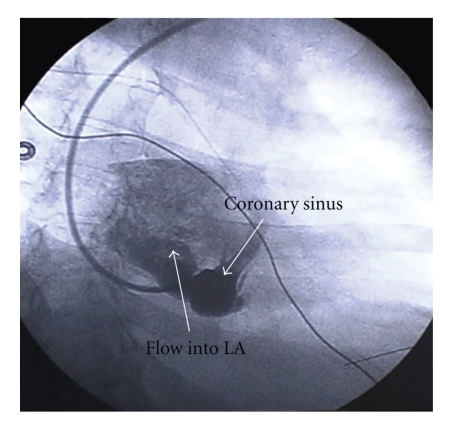
Coronary Sinus Venogram demonstrating an enlarged CS with communicating vessel into LA.

With this presumptive diagnosis, two attempts to cannulate the coronary sinus for angiography were made. Initial attempt from the right femoral vein was not successful partly due to a thebesian valve at the coronary sinus ostium; hemodynamic profile at this point, after 25 lbs of volume removal, showed RA 25, PA 40/15, PCWP 12 mmHg. A repeat attempt was made 2 days later in the electrophysiology suite, via the right internal jugular approach, with adequate engagement of the coronary sinus. During this attempt, the patient's respiratory status worsened and she was intubated in the suite. Venogram of the coronary sinus revealed a large coronary sinus that appeared to have two outlets, one to the RA and another to the LA. Direction of contrast flow was entirely from the RA to the LA (Figure 5).

The patient was then referred to surgery where a communication between the CS and the LA was repaired and the tricuspid valve repaired with an annuloplasty ring. The right ventricle was noted to be “entirely infarcted.” After surgical correction, her pO_2_ was 123 on 100% FiO_2_ with O_2_ saturations at 99%. Postoperatively, central venous pressures remained elevated at 20 mmHg. Despite initial hemodynamic instability, her major difficulty involved inability to wean from the ventilator complicated by pneumonia and eventual gram negative sepsis. After approximately 20 days, she underwent a tracheostomy and was discharged to a long-term acute care facility, where she was successfully decannulated 1 month later.

## 2. Discussion

Communication between the LA and CS can sometimes be confused with an atrial septal defect [1], with predominantly a left to right shunt. Although, enlargement of the CS may occur in different pathologic states such as congestive heart failure, in the current case, it appears to be the result of an anomalous connection between the CS and LA. Mantini et al. [2] classified cases of enlarged coronary sinus ostium into 2 groups based on the characteristics of the left-to-right shunt. With a high pressure left-to-right shunt, a coronary arteriovenous fistula is involved, while with a low pressure left-to-right shunt, an anomalous communication of the coronary sinus with the LA or with the pulmonary venous system would be implicated [3]. Communication of the coronary sinus with the LA may be either (1) indirect through a bridging vein between the two structures or (2) direct by way of an opening between the lateral aspect of the coronary sinus and the left atrial cavity Mantini et al. [2]. We observed the latter in our patient as evidenced by cardiac CT and coronary sinus venogram. Possible etiologies for this defect include congenital [4], cardiac trauma or iatrogenic causes; the patient did not have a history of such trauma or CS cannulation at time of presentation. 

**Figure 6:**
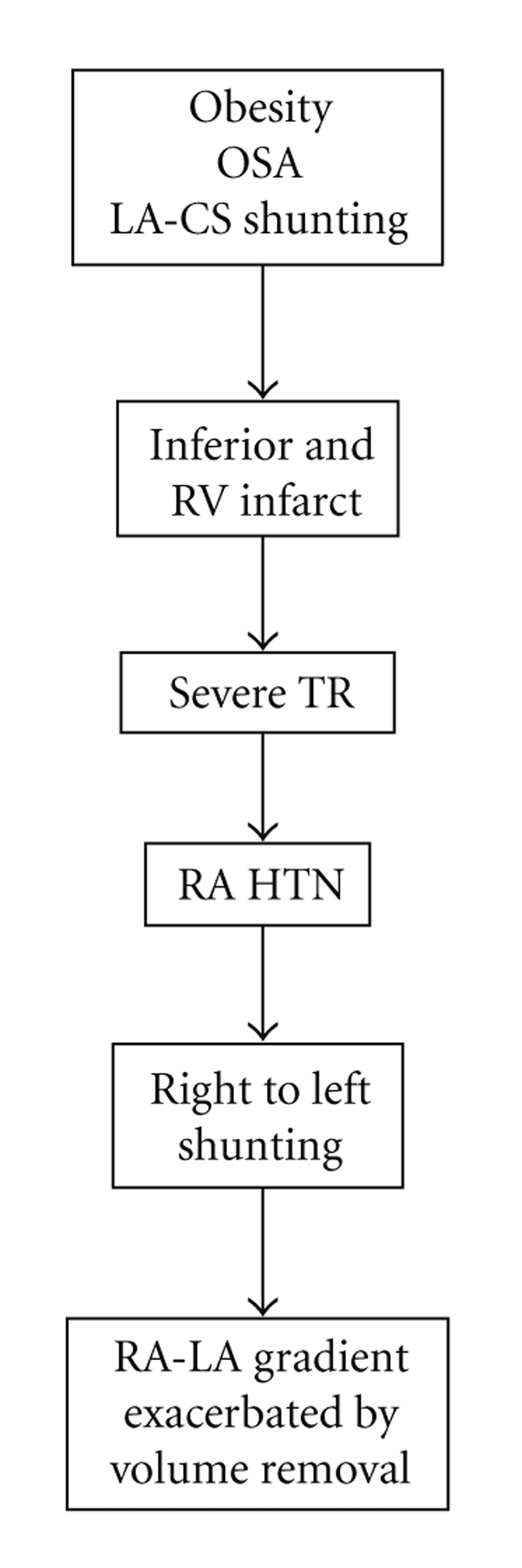
Flow diagram indicating patients hemodynamic status resulting in severe left-to-right shunting.

 In this patient, her presumed congenital abnormality remained undetected, likely with underlying left to right shunting until, with progression of her right ventricular dysfunction, tricuspid regurgitation, and right atrial hypertension, the direction of shunting was reversed, resulting in severe hypoxia. We also noted worsening of her hypoxia with aggressive volume removal. We hypothesized that ultrafiltration appeared to reduce the LA pressure (17 to 12 mmHg) to a greater extent than her RA pressure (25 mmHg and unchanged), possibly due to her unremitting tricuspid regurgitation. This differential effect on the atrial hemodynamics would be expected to lead to an increase in the right-to-left shunting. (Figure 6). 

 In summary, a CS anomaly may occur as an isolated anomaly with no functional clinical significance; however, hemodynamic perturbations can lead to severe right-to-left shunting and hypoxia. Recognition of this abnormality in the differential diagnosis of shunting in either direction at the atrial level can be confirmed with high quality CT angiography and coronary sinus venogram.
